# Comparative Zymocidial Effect of Three Different Killer Toxins against *Brettanomyces bruxellensis* Spoilage Yeasts

**DOI:** 10.3390/ijms24021309

**Published:** 2023-01-09

**Authors:** Alice Agarbati, Maurizio Ciani, Semih Esin, Monica Agnolucci, Fabio Marcheggiani, Luca Tiano, Francesca Comitini

**Affiliations:** 1 Department of Life and Environmental Sciences, Polytechnic University of Marche, Via Brecce Bianche, 60131 Ancona, Italy; 2 Department of Translational Research and New Technologies in Medicine and Surgery, University of Pisa, Via San Zeno 37, 56123 Pisa, Italy; 3 Department of Agriculture, Food and Environment, University of Pisa, Via del Borghetto 80, 56124 Pisa, Italy

**Keywords:** wine microbiology, biocontrol, killer yeasts

## Abstract

Three killer toxins that were previously investigated, one excreted by Kluyveromyces wickerhamii and two by different strains of Wickerhamomyces anomalus, were produced at the pilot scale, lyophilized and characterized, and the formulates were assessed for their zymocidial effect against Brettanomyces bruxellensis spoilage yeast. A comparative analysis allowed the evaluation of the minimum inhibitory concentration (MIC) against a sensitive strain. Fungicidal and fungistatic concentrations were used to evaluate the cytocidal effect using a cytofluorimetric approach that confirmed the lethal effect of all lyophilized formulates against *B. bruxellensis* spoilage yeasts. Moreover, the potential killer toxins’ cytotoxicity against human intestinal cells (Caco-2) were evaluated to exclude any possible negative effect on the consumers. Finally, the effective lethal effect of all three lyophilized killer toxins toward *B. bruxellensis* sensitive strain were tested. The results indicated that all of them acted without dangerous effects on the human epithelial cells, opening the way for their possible commercial application. In particular, D15 showed the lowest MIC and the highest activity, was evaluated also in wine, revealing a strong reduction of *Brettamonyces* yeast growth and, at the same time, a control of ethyl phenols production.

## 1. Introduction

Killer toxins are proteins or glycoproteins able to bind receptors on the surface of the specific target microorganisms [1]. Their lethal action can be expressed through different and specific modes of action. The killer trait is widespread among the killer toxins’ activity, and it has been reported in over a hundred yeast species, several of them with a potential application in various industrial sectors, such as food, pharmaceutical, agricultural and fermentation, due to their antagonistic activity against pathogens [2]. In winemaking, killer toxins are interesting biocontrol tools to enhance the wine quality by removing spoilage yeasts with consequent reduction of the use of chemical preservatives [3,4].

Several killer toxins secreted by different non-*Saccharomyces* yeast strains are active against a wide range of spoilage yeasts, including *Dekkera*/*Brettanomyces* yeasts, responsible for unpleasant odors produced during wine fermentation, aging and storage, that affect the quality of the final product. Considering the growing interest in reducing sulfur dioxide during winemaking, the killer toxins could represent an interesting alternative.

The potentially effective killer toxins are those that do not negatively interact with *Saccharomyces cerevisiae* fermenting yeasts, nor with malolactic bacteria correlated to optional malolactic fermentation.

*Kluyveromyces wickerhamii*, *Pichia anomala*, *Pichia membranifaciens* (the last two species recently reclassified as *Wickerhamomyces anomalus*) and the filamentous fungus *Ustilago maydis*, have recently been described as possible alternatives to chemical treatments, to prevent or reduce *Brettanomyces* spoilage risk [5].

The killer activity of these toxins was found to be either fungistatic or fungicidal, depending on their concentration [6]. Similar results were found by Santos et al. [7] for the high concentration of the PMKT2 killer toxin, leading to an increasing mortality rate in the three *B. bruxellensis* strains tested [6]. Moreover, lower concentrations of the killer toxin from *U. maydis* significantly reduced the amount of 4-ethylphenol produced by *B. bruxellensis*, indicating that, in addition to the growth inhibition observed for high concentrations of killer toxins, its small amounts (100 AU) can control flavor defects in wines [8].

More recently, the killer toxins CpKT1 and CpKT2, isolated from the wine yeast *Candida pyralidae*, were active and stable under winemaking conditions and appeared to be able to cause cell membrane and cell wall damages in *B. bruxellensis*.

Villalba and coworkers [9] recently identified and partially characterized a new killer toxin from *Torulaspora delbrueckii* with wide biocontrol spectrum against *B. bruxellensis*, *Pichia guilliermondii*, *Pichia manshurica* and *W. anomalus* wine spoilage. 

Nowadays, the production of natural antimicrobial compounds, including killer toxins, is a fundamental topic for the modern biotechnology industry [10]. Although the market for traditional industrial antimicrobials is expanding, the research emphasis on biotech efforts is generating a growing demand for natural alternatives with lower impact on the consumer’s health. The advent of genetic engineering, together with cutting-edge technological processes, is facilitating the large-scale production of such molecules, including proteins with antimicrobial action or industrial enzymes produced naturally only in small quantities [11]. While this development is particularly evident with regards to the production of therapeutic proteins, in the food sector research it is poorly translated into industrial applications, probably because the level of protein downstream processing depends on its intended application [12]. The proteins that are potentially interesting in the industry are those produced in bulk and generally requiring little downstream processing and, therefore, relatively crude and inexpensive preparations.

This work is put in such context: a simple technological process of industrial production at the pilot scale, followed by easy downstream steps, to verify the antimicrobial efficacy of killer toxins previously purified and characterized at the laboratory scale, for the control of *B. bruxellensis* spoilage yeasts in winemaking [13,14,15,16].

In particular, three different killer toxins, two excreted by two different *W. anomalus* strains and one secreted by *K. wickerhamii* yeast, were produced at the pilot scale, semi-purified and lyophilized, following an industrial production step [13,14,15,16].

Three lyophilized formulations were separately assayed in vitro and in wine to compare their cytocidal activity and their effect on *B. bruxellensis*. The safety aspects in human intestinal cells were also evaluated.

## 2. Results

### 2.1. Killer Toxins Industrial Production and Lyophilization

The bioreactor cultivation of *W. anomalus* DiSVA2 and DiSVA671, as well as the *K. wickerhamii* DiSVA15 strains, confirmed the production of Pikt, WA18 and Kwkt, their respective native killer toxins previously described and characterized (see Materials and Methods section)

**Figure 1:**
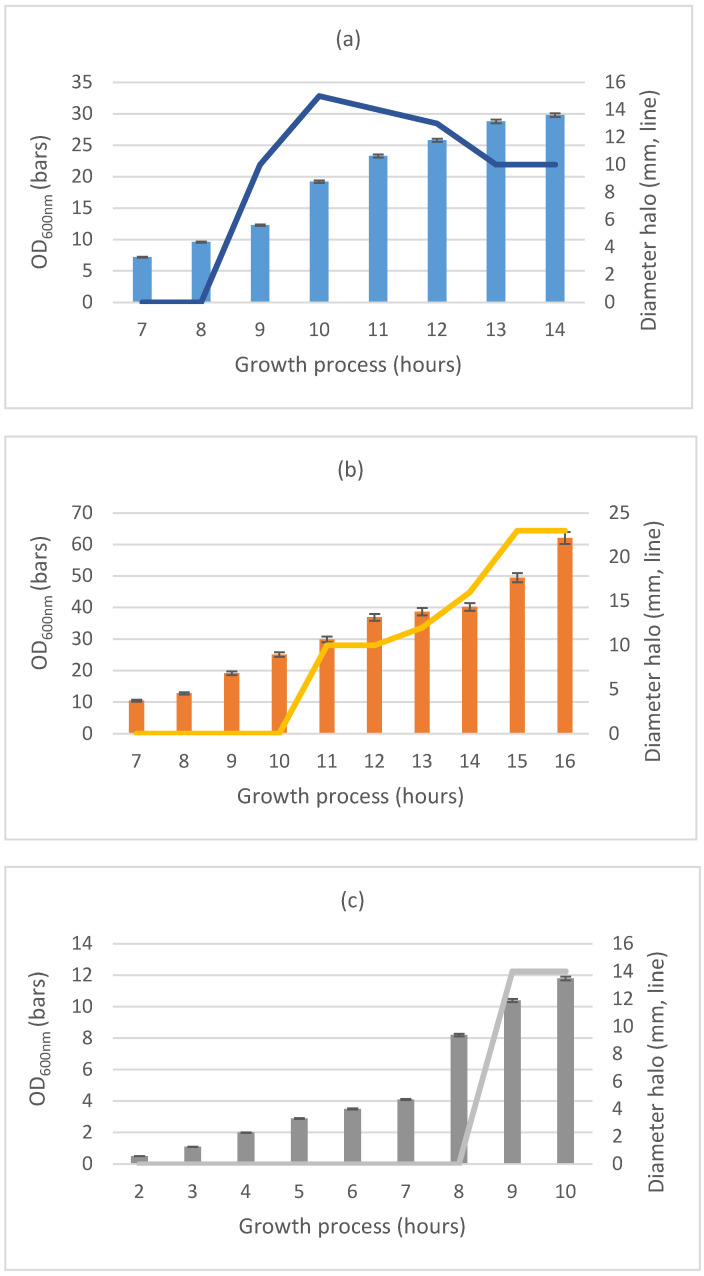
Ki netics of growth and killer toxin activity of (**a**) *Wickerhamomyces anomalus* DiSVA2, (**b**) *Kluyveromyces wickerhamii* DiSVA15 and (**c**) *Wickerhamomyces anomalus* DiSVA671, tested against *Brettanomyces bruxellensis* sensitive strain. Growth was measured as OD600 nm, toxin production was evaluated as size of the inhibition halo (in mm, considered 8 mm the wall diameter) in well test assays. Colored bars (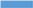), (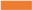), (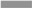) indicate values of OD600 nm for *W. anomalus* DiSVA2, *K. wickerhamii* DiSVA15 and *W. anomalus* DiSVA671. Similarly, lines (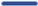), (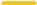), (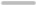) indicate the values of diameter. Microbial growth (bars) was reported as mean values ± standard deviations.

During the pilot scale conditions, the productivity and yields of killer toxins were increased. Indeed, the maximal lab-scale production (in flasks) of each killer toxin was delayed by 6–10 h, if compared to the bioreactor production. Figure 1 showed the growth of each yeast and the relative production kinetics of each toxin. The maximum concentration of the toxin produced by *W. anomalus* DiSVA2 was obtained after 10 hours of incubation, that of *K. wickerhamii* DiSVA15 around the 15^th^ hour and finally *W. anomalus* DiSVA671 produced greatest amount of toxin at the 19^th^ hour.

For each killer toxin, a fermented broth ten-fold concentrated (named broth 10 ×) and partially purified, was obtained by ultrafiltration using tangential flow membranes, as a single downstream step.

The obtained lyophilizates named D2 (from *W. anomalus* DiSVA2), D15 (from *K. wickerhamii* DiSVA15) and D18 (from *W. anomalus* DiSVA671), obtained with yield of 12.3, 22.4 and 55.9 g/L, respectively, were then solubilized in order to be subsequently used After various tests carried out considering the physical solubilization and the economic implications relating to their further use in relation to the production costs, the dose of 100 g/L was chosen as the maximal stock concentration.

The solubilization test of stocks and, therefore, their antimicrobial efficacy was carried out in water, in 0.1 M citrate-phosphate buffer pH 4.4, and wine. The results obtained after well test assays indicated that no differences in the solubility and antimicrobial activity in D2, D15 and D18 in all preparations were observed. Thus, sterile, distilled water was utilized. The D2 lyophilizate showed an increment of 2 mm in the inhibition halo, if compared with the relative ultrafiltered, cell-free supernatant. For D15, the same halos were obtained by testing both the ultrafiltered cell-free supernatant and the lyophilized preparation, while a greater increment of 4 mm was observed for the D18 lyophilized preparation halo (Table 1). 

The evaluation of the long-term stability of all lyophilizates, both in powder and loose form, showed no significant variations of killer activity for up to nine months at 10 °C. 

The solubilization of stock lyophilizates at the tested concentrations (100 g/L) showed very low residual sugars, while the protein component increased significantly when compared with the partially concentrated broth, reaching comparable values for all three preparations (about 45 g/L), actually ten-fold higher taking into account the stock dilution factor. The slight increase in the antimicrobial activity of the lyophilizates was consistent with the results of the protein since the halo diameter was in logarithmic function of the concentration of active compounds (Table 1). An exception was shown by the D2 lyophilized sample, where the diameter of the inhibition halo was already in the limit of sensibility of the well test method. 

### 2.2. Minimum Inhibitory Concentration (MIC) of the Three Killer Toxins

The results of in vitro MIC determinations showed that D15 was the most active against *B. bruxellensis* with the lowest MIC value of 0.05 mg/mL. D2 also displayed significant in vitro effects but with a double MIC value of 0.1 mg/mL. The highest MIC value obtained against the sensitive strain was exhibited by D18. 

The measures of the inhibition halo, corresponding to increasing concentrations of each killer toxin, were reported in Table 2. Concerning D2 and D15, a progressive decrease in the antimicrobial action was observed (progressive decrease in the inhibition halos). Regarding D18, after the first dilution, showing a small halo, the activity seemed to disappear. This was probably due to the lower initial killer activity that could not be detected by the sensitivity of the well test method. 

**Figure 2:**
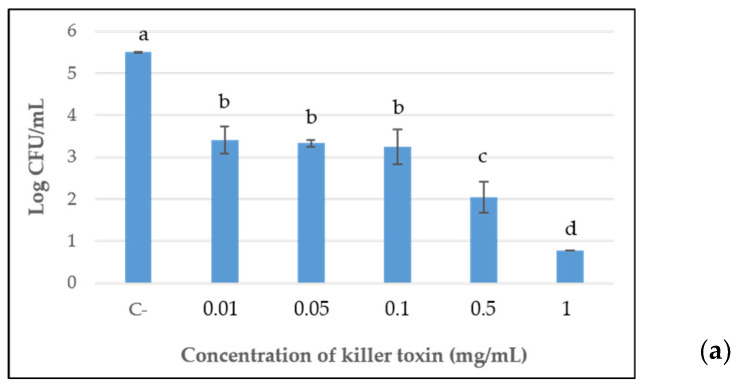

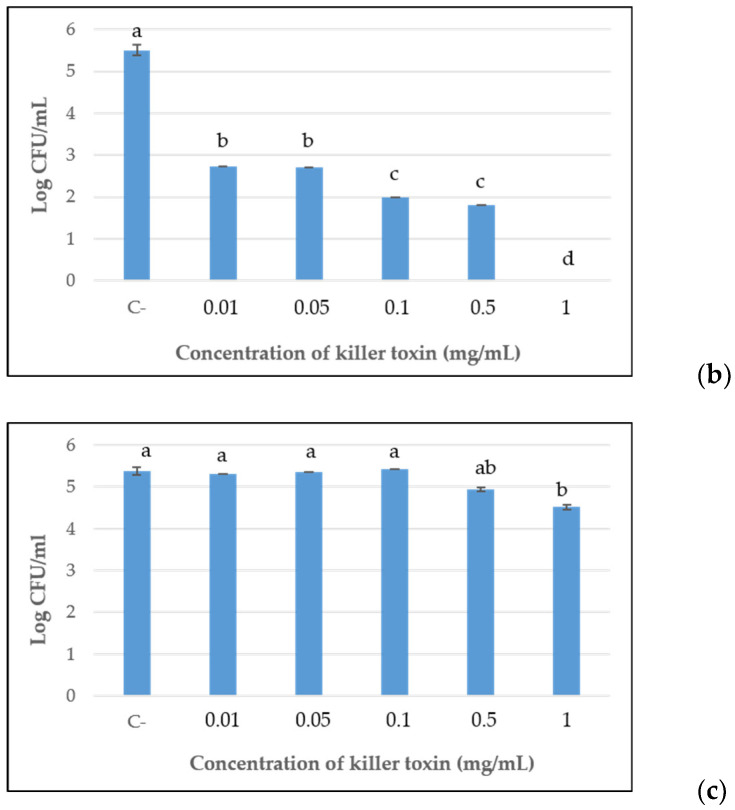
Viable cell counts of *B. bruxellensis* after 96 h of incubation with different concentration of killer toxins. (**a**–**c**) graphs represent the sensitive yeast incubated with D2, D15 and D18 toxins, respectively. C- represents the negative control where no killer toxin was added. Data mean ± standard deviations (error bars) and values showing different superscript letters (^a,b,c,d^) within each graph indicate significant differences between the treatments, according to Duncan test (*p* < 0.05).

Furthermore, the killer toxins, at each concentration previously assayed for MIC determinations, were tested for their effective lethal activity toward the sensitive strain, after 96 h of incubation, by viable cell counts assay. The results reported in Figure 2, confirmed the progressive reduction of antimicrobial activity in a dose-dependent manner of the D2 and D15 lyophilized preparations up to 0.1 mg/mL and 0.05 mg/mL, respectively. After that, a constant significant antimicrobial action was shown. For D15, no cultivable cells were detected at the maximum concentration tested (1 mg/mL), while at 0.5 mg/mL and 0.1 mg/mL, the concentrations showed similar results with a reduction of about 3.5 log orders of magnitude, in comparison with the control. A significant containment of the development of *B. bruxellensis* was also detected for the doses of 0.05 and 0.01 mg/mL. The same trend was observed for toxin D2, although with less effective containment of yeast growth than D15.

Differently, concerning D18, only the maximal concentration of 1 mg/mL significantly reduced the yeast development compared with the control, by approximately 1 log order of magnitude, while testing 0.5 mg/mL concentration, only a slight reduction was shown.

### 2.3. Cytofluorimetric Assay

Imaging flow cytometric analyses were performed in order to detect changes induced by the D2, D15 and D18 treatments to sensitive *B. bruxellensis* cells, previously grown in YM liquid medium and treated for 96 h with each lyophilized killer toxin, separately.

**Figure 3:**
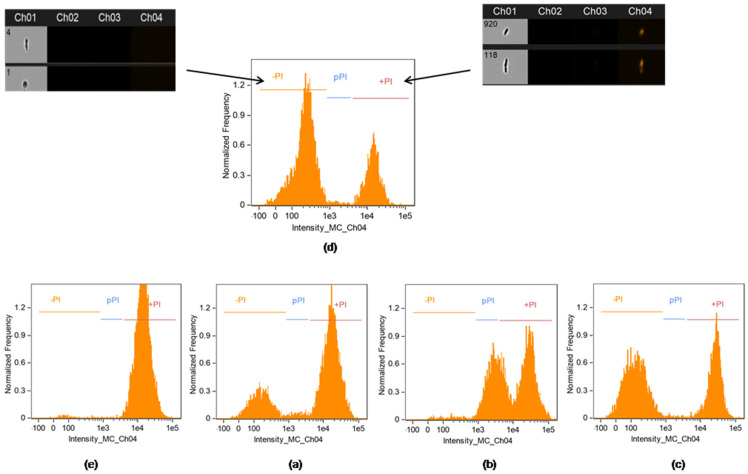
Imaging Cytofluorimetric assay. The images represent the distribution of the *B. bruxellensis* population in live cells (-PI gate), partially PI positive (pPI gate) and dead cells (+PI), after 96 h of incubation with 1 mg/mL of each D2 (**a**), D15 (**b**) and D18 (**c**). Negative control with live cells without any killer toxin (**d**). Positive control with ethanol-killed cells (**e**).

As reported in Figure 3 and the relative data summarized in Table 3, in untreated controls, the large majority of the cells (71%) was composed of live cells, while 28% of the cells were clearly propidium iodide (PI) positive (dead cells). The validity of the treatment has been assessed using a positive control, where cells were incubated in ethanol. In these conditions, the percentage of live cells decreased to 0.76%, while PI positive cells accounted for 98.7%. The relative efficacy of the toxins tested is summarized in Table 3, showing the following degree of cellular toxicity: the highest percentage of PI positive cells was detected in the D2 toxin (73.3%), followed by the D15 toxin (64.1%) and D18 (40.7%). Notably, in sample D15 (Figure 3b), the percentage of live cells was particularly decreased, and, uniquely in these samples, the partially PI positive cells increased considerably to 30.1% compared to D2 2.7% and D18 0.78%.

The D2 and D15 (Figure 3a,b) lethal effect was significantly higher when compared to D18 (Figure 3c) with differences more pronounced among live and dead cell populations. The negative control (Figure 3d) was also evaluated after 96 h of incubation, similarly to those of lyophilized formulations, thus the resulting dead population represented the fraction of *B. bruxellensis* physiologically dead, after 4 days of incubation, probably due to the lack of nutrients. 

The percentage of the relative mortality of the cells evaluated with the flow cytometer was compared with the effective number of cells culturable by viable plate counts. Compared with the initial inoculum (Log 4 CFU/mL), the *B. bruxellensis* cells after 96 h of incubation reached a value of Log 6.5 CFU/mL. This value was considered equal to 100% of the live cells, as reported in Table 3, right columns. Similarly, the results of the viable counts of toxin-treated sensitive cells were reported as the relative percentage of the control (100%). The highest concentration tested showed an evident fungicidal activity of all lyophilized formulations towards the sensitive strain.

### 2.4. Cytotoxicity against Human Intestinal Cells (Caco-2)

**Figure 4:**
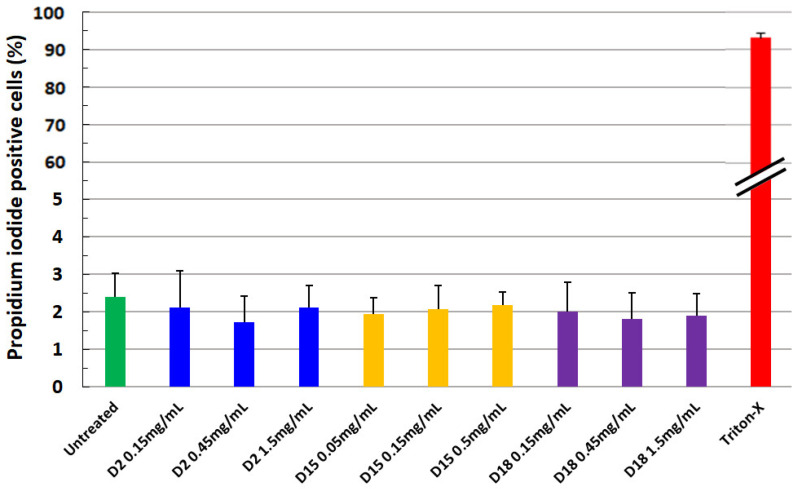
Cytotoxic activity of the three killer toxins (D2, D15, D18) against human intestinal cells (Caco-2 cells) measured as incorporation of propidium iodide (PI) into DNA of dead cells. Mean ± SEM of four experiments in duplicates.

The cytotoxic activity of the three killer toxins was tested in vitro against the human intestinal cell line Caco-2. As depicted in Figure 4, a very low (less than 3%) cytotoxic activity of the killer toxins was observed following 24 h exposure in the experimental conditions.

### 2.5. D15 Lyophilized Formulate Tested in Wine

**Figure 5:**
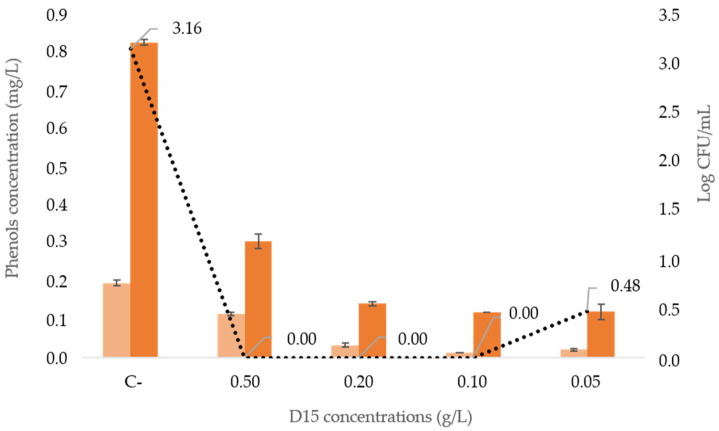
Viable cell counts and relative phenols determinations in spontaneous contaminated wine incubated for 60 days. Different dilutions starting from 100 g/L of D15 lyophilized stock were assayed. (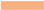), (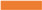) and (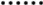) represent 4-ethyl guaiacol and 4-ethyl phenol concentrations and Log CFU/mL, respectively. C- represent the negative control, where no killer toxin was added. Phenols determinations are reported as mean value ± standard deviations (error bars).

Starting from the 100 g/L stock of the most effective lyophilizate D15, different dilutions of 0.5 g/L, 0.2 g/L, 0.1 g/L and 0.05 g/L were tested in a spontaneous contaminated red wine (concentration *Brettanomyces* spp., about Log 3 CFU/mL). The efficacy of the D15 formulation was evaluated both by the viable cell counts and through the measurement of the phenols produced after 60 days of D15 addition. The results reported in Figure 5 clearly showed that all concentrations tested inhibited the growth of *Brettanomyces* yeasts, with the exception of the lowest dilution, where a slight *Brettanomyces* population survived. Similarly, ethyl phenols significantly decreased if compared with the untreated control (C-).

## 3. Discussion

The native antimicrobial compounds applied in food and beverages as preservatives combine the protection of the final product with extended shelf life and consumer safety. 

The potential roles of many naturally occurring antimicrobial compounds, such as bacteriocins, nisin, plant essential oils, chitosan, have been widely studied and are reported to be effective against spoilage and pathogenic microorganisms. Few of these compounds are commercially available and used in food processing, since their efficacy, consumer acceptance and regulation are not well defined [17].

In the enological field, although there are numerous genera and species of yeasts able to produce effective killer toxins against the development of *Brettanomyces* spp. spoilages, the commercial formulation of such characterized molecules remains a prerogative of the applied studies [5,18].

The potential uses proposed for yeast killer toxins have been limited by the recognized skills of these toxins [19]. The suggested applications are largely limited by the physical and chemical conditions fitting with the stability of killer toxins, mainly low pH and temperature values. 

Although the use of killer yeasts is effective at limiting the risk of the onset of *Brettanomyces* spp., their antimicrobial activity could represent a preventive action during alcoholic fermentation, but it is not suitable to solve in highly compromised situations or to be used in the advanced stages of production when the yeast inoculum is not easy to be performed. By contrast, the formulation of purified products to be added at concentrations able to guarantee greater efficacy, associated with a higher similarity with conventional chemical analogues, is limited due to the high production costs and low stability of the active compounds [6,20,21].

Recently, novel agents based on natural antimicrobials (chitosan, essential oil compounds) or metal nanoparticles (silver nanoparticles) have been suggested to replace or reduce the use of SO_2_ in winemaking. Although these strategies improve the antimicrobial characteristics of essential oils, the high production costs, related to the complex down-stream steps, limits the commercialization of these natural antimicrobials [22]. 

In the present work, the formulated ready-to-use killer toxins, denominated D2, D15 and D18, were characterized and compared for their zymocidial effect against *Brettanomyces* yeasts, a most relevant spoilage agent in winemaking. 

The determination of viability using image-based flow cytometry, coupled with PI staining, allowed for the precise determination of live (-PI) and dead (+PI) cells. Compared with other flow cytometric determinations, the use of these instruments decreases potential artifacts associated with the erroneous classification of debris, with the images of each event acquired and used for the separation of the populations [23,24,25]. In this work, the data highlighted a remarkable increase in the percentage of dead cells in the D2-treated samples, while a lower effect was observed in the D18 samples. The samples treated with D15 showed a particular distribution: while the percentage of dead cells did not increase remarkably compared with other samples, the live (-PI) population was basically lowered to a residual 2.06% population, highlighting the particular toxicity of this compound. This behavior, in the experimental time frame, led a very evident shift of the distribution toward higher levels of PI fluorescence, stressing the particular cytotoxicity of the killer toxin D15.

The viable counts, carried out after 96 h of inoculation of the lyophilizates, allowed us to validate the actual death of the sensitive cells observed using imaging flow cytometric determination, in order to exclude a possible state of VBNC (viable but not culturable), typical of the *Brettanomyces*/*Dekkera* spp. strains [26]. Indeed, in the VBNC state, as a result of some treatments with low SO_2_ doses or chitosan, the production of 4-ethylphenol stopped and the possible wine conditions changed (e.g., concentration of molecular SO_2_ decreases), so the spoilage yeasts could theoretically regrow and re-infect the wine [27,28]. Here, the results of D15 in the real condition in wine confirmed the long-term control of *Brettanomyces* yeasts and, at the same time, the control on the ethyl phenols production.

Another fundamental characterization of antimicrobial compounds for a possible application concerns the evaluation of their cytotoxicity. Here, the exclusion of a cytotoxic effect by the killer toxins tested, assessed on human intestinal epithelial cells [29], suggests their possible safe use in food production after the necessary validation of the formulations. Therefore, in agreement with the safety of the yeast killer toxin on animal systems [30], our results are compatible with the hypothesis that, in the tested concentrations, the three killer toxins should not represent a potential health risk for human consumption.

In conclusion, similarly to the antiseptic action performed by sulfur dioxide, the use these three killer toxins pilot-scale produced and lyophilized could represent an effective natural strategy for controlling the onset or the proliferation of *Brettanomyces*/*Dekkera* spoilage yeasts, as the possible substitution of chemical additives. Depending on the concentration, all preparations greatly reduced the *B. bruxellensis* populations; in particular, D15 was also efficient during the industrial winemaking step, revealing a healing power even when the *Brettanomyces* spp. infection was already established. Therefore, D15 represents a strong candidate as a natural antifungal agent for the wine industry.

## 4. Materials and Methods

### 4.1. Killer Toxins Production and Characterization

Three killer toxins, named Pikt, Kwkt and WA18, were previously described and characterized, and the main features are shown in Table 4. They were produced by three different yeast strains coming from the Yeast Collection of the Department of Life and Environmental Sciences (DiSVA) of the Polytechnic University of Marche (Italy), such as *Wickerhamomyces anomalus* DiSVA2, *Kluyveromyces wickerhamii* DiSVA15 and *Wickerhamomyces anomalus* DiSVA671, for Pikt, Kwkt and WA18, respectively. On the basis of the results previously obtained at laboratory scale, here the killer toxins were produced at the pilot scale using bioreactors, partially purified and then lyophilized. 

### 4.2. Set Up of Pilot Scale Killer Toxins Production

On the basis of the preliminary results, here the pilot scale production (300 L) of the three killer toxins was set up. The medium used has the following composition: yeast extract 10 g/L; malt extract 5 g/L and casein peptone 5 g/L, buffered to pH 4.4 with 0.1 M citric acid/dibasic sodium phosphate. The glucose concentrations were 30 g/L for Pikt and 40 g/L for both Kwkt and WA18, to avoid the presence of residual sugars at the end of the process.

Fresh cultures of the yeasts *K. wickerhamii* DiSVA15, *W. anomalus* DiSVA2 and *W. anomalus* DiSVA671 were used to inoculate the medium (equal to 5% of the final volume of the growth process) for the production of each killer toxin. The growth conditions for the process were: pH 4.4, temperature 25 °C, pO_2_ 20% and stirring 250 rpm. The process was followed for 16 h, 10 h and 9 h for Kwkt, Pikt and WA18, respectively. At the end of the process, the supernatant of each culture was microfiltered (Ø 0.2 µm cut-off) and the acellular broth was subjected to ultrafiltration (BioFlex 50, Schleicher & Schuell, Whatman GmbH, GE Healthcare, Dassel, Germany) using a membrane with 10 KDa cut-off, with the purpose to concentrate the broth 10-fold, then consequently concentrate the protein killer. Afterwards, each concentrated broth underwent a dialysis process (4-fold) using the same ultrafiltration system and 0.1 M citric acid/dibasic sodium phosphate buffer, obtaining a broth 10 × partially purified for each toxin. The purpose was to remove the residual sugars and unnecessary proteins/metabolites to obtain a greater purification of the concentrated killer protein.

The cell-free supernatants at the end of each growth process and the broths 10 × partially purified were tested for the presence of killer activity against a common wine spoilage yeast, *Brettanomyces bruxellensis* DiSVA 46 (Yeast Collection of the Department of Life and Environmental Sciences of the Polytechnic University of Marche, Italy), through the well test screening method, as described by Comitini et al. [13]. Briefly, an aliquot of the sensitive *B. bruxellensis* DiSVA46 pure culture was suspended in sterile water to obtain a concentration of about 10^6^ cells/mL. In total, 1 mL of this suspension was put inside the 90 mm Petri dish with 20 mL of buffered Malt Agar (2.7% malt extract, 1.8% agar, buffered to pH 4.4 with 0.1 M citric acid/dibasic sodium phosphate) still liquid, and well-distributed. Prior to the agar solidification, sterile steel wells, with 6 mm of diameter, were placed on the agar. Left to solidify the medium, the wells were removed with sterile forceps with the aim to create a space then filled with 70 µL of each killer toxin suspension.

The plates were incubated at 22 °C for about five days and the killer activity of the lyophilized toxins against the sensitive *B. bruxellensis* strain was evaluated by measuring the diameter of the halo around the wells, corresponding to the zone of growth inhibition of the sensitive yeast [32].

Moreover, the glucose content and total protein content of all cell-free supernatants and broths 10 × partially purified were determined. The glucose concentration was determined using a specific enzymatic kit (Megazyme International, Ireland, Wicklow, Ireland), while the total protein content was expressed as the gallic acid equivalents, following the method described by Singleton et al. [33].

### 4.3. Lyophilization Step

The broths 10 × partially purified, concerning each Pikt, Kwkt and WA18 killer toxins, were subjected to the lyophilization process, obtaining three preparations named D2, D15 and D18, respectively. The lyophilization was carried out frozen for each broth 10 × partially purified at −20 °C, then transferred to the lyophilizer for 48 h, temperature −53.2 °C, pressure 1 mbar.

The lyophilized preparations have been assayed for their killer activity against a common spoilage yeast of wine, *B. bruxellensis* DiSVA46, following the well test screening method, as described in Section 4.2. The lyophilizates D2, D15 and D18 were subjected to a solubilization test and, therefore, their antimicrobial efficacy was evaluated. In total, 100 g of each lyophilizate was rehydrated in 1 L of H_2_O, 0.1 M citrate-phosphate buffer (pH 4.4) or wine and sterilized by 0.45 µm pore-size filters, in order to establish the possible differences in solubilization and killer activity, depending on the solvent used. 

The well test plates were incubated at 22 °C for about five days and the killer activity of the lyophilized toxins against the sensitive *B. bruxellensis* strain was evaluated by measuring the diameter of the halo around the wells, corresponding to the zone of growth inhibition of the sensitive yeast [32]. The glucose and protein content of each lyophilized product (100 g/L in water) were determined as described above.

### 4.4. Determination of Minimum Inhibitory Concentration (MIC) of the Three Killer Toxins

The determination of the MIC regarding the three killer toxins against sensitive *B. bruxellensis* was carried out following the procedure reported by the Clinical and Laboratory Standards Institute guidelines [34], with some adaptations. Briefly, a 96-well plate was filled with Yeast Mold broth (yeast extract 3 g/L, malt extract 3 g/L, peptone 5 g/L, glucose 10 g/L) buffered to pH 4.4 with 0.1 M citric acid/dibasic sodium phosphate and inoculated with 1 × 10^4^ cell/mL of *B. bruxellensis* DiSVA46 fresh culture. Then, five different concentrations of each killer toxin were tested, ranging from 1 mg/mL to 0.01 mg/mL. Each test was performed in duplicate and the medium inoculated with *B. bruxellensis* DiSVA46, and without the toxins was used as a control. The 96-well plate was incubated at 22 °C for 96 h. Then, viable cell counts were carried out to determine the presumed reduction of yeast viability due to the killer toxin activity. Hence, 100 µL of suspension from each microplate well was spread on Yeast Mold Agar plates and compared with the control. 

### 4.5. Citotoxicity of Killer Toxins for B. bruxellensis-Imaging Cytofluorimetric Assay

The three lyophilized killer toxins (D2, D15, D18) were tested through cytofluorimetric assay, with the aim to compare their cytotoxicity against *B. bruxellensis* DiSVA46. Here, the sensitive yeast was cultured in the Yeast Mold broth buffered to pH 4.4 with 0.1 M citric acid/dibasic sodium phosphate for 48 h. The fresh culture was centrifuged and washed twice with 0.9% NaCl and used to obtain flasks containing a cell suspension of about 1 × 10^4^ cell/mL in 0.9% NaCl and inoculated with 1 mg/mL of each toxin. The flasks were incubated at 22 °C for 96 h prior to imaging cytofluorimetric assay. A negative control was conducted without the killer toxins (live cells), while 80% ethanol was added to ensure the obtaining of dead cells (positive control).

The cytotoxicity of *Brettanomyces* was evaluated using Imaging Flow Cytometer (IFC) FlowSight (Luminex, Austin, TX, USA) with propidium iodide (PI) probe.

The (IFC) assay was performed with 300 000 yeast cells obtained by centrifugation at 600 g × 6 min of 10 mL of *Brettanomyces* at 30 000 cells per mL. After that, the yeast pellet was resuspended in 100 µL of PBS and cell suspension was stained with 10 µL of PI (1 mg/mL), before acquisition was performed by FlowSight.

The samples were excited with a 488 nm laser with the following laser powers: 488 nm 5.00 mW; 785 nm 3.75 mW. The imaging flow cytometry analysis was performed on 8000 focused and singled cells gates (R2) set up in aspect ratio and area dot plot in the Channel 1 (Ch1) mask. Within the R2 gate, the emission of PI was reported as the Fluorescence intensity of Channel 4 (Ch4).

After the acquisition phase, before analyzing the live and dead cells, the raw data files (rif) were processed by the IDEAS software (Luminex, USA) to discriminate the *Brettanomyces* population from cellular debris.

Arbitrary gates were defined in the control untreated cells (Figure 2d) in order to identify propidium negative cells (live) and propidium positive cells (dead). The histogram of distribution also shows an intermediate population with partial permeability to PI that accounts for the 100 − (% live + % dead). This partial positivity to PI is suggestive of potential apoptotic processes.

Moreover, to confirm the cytofluorimetric results, it was also carried out the microbiological assay by viable plate counting.

### 4.6. Cytotoxicity Assay against Human Intestinal Cell Line Caco-2

The cytotoxic potential of the three lyophilized killer toxins (D2, D15, D18) was tested against the human intestinal cell line Caco-2 derived from human colorectal adenocarcinoma. To this aim, the cells were seeded in 96 well plates at a density of 2 × 10^4^ cells/well in high glucose DMEM added with 2 mM L-glutamine, 1% non-essential amino acids and 10% FBS. The cells were incubated for 24 h at 37 °C in a humidified atmosphere containing 5.5% CO_2_ to reach approximately 90 to 100% confluence. Following a wash to remove non-adherent cells, adherent cells were added with various concentrations of the three killer toxins (D15: 0.05, 0.15, 0.5 mg/mL; D2 and D18: 0.15, 0.45, 1.5 mg/mL), with the complete medium only (negative control), or with 0.01% Triton X-100 in PBS (positive control for cytotoxicity) in the humidified atmosphere containing 5.5% CO_2_ and incubated for 24 h at 37 °C. After incubation, the adherent cells (previously washed twice with warm PBS) were detached with Trypsin/EDTA treatment. The pellets were washed with PBS (8 min 400× *g*) and were resuspended in the PBS. Following 5 min staining with 1 µg/mL PI at room temperature, 50,000 events were acquired ungated by using a flow cytometer (BD Accuri C6, BD biosciences, Franklin Lakes, NJ, USA). The percent of PI positive, dead Caco-2 cells weas calculated by computer-assisted analyses (BD Accuri C6 software, BD Biosciences). Four independent experiments with duplicates were carried out.

### 4.7. Application of D15 in Wine

Wine naturally contaminated with *Brettanomyces* yeasts was treated with different concentrations of D15. The 4-ethylphenol and 4-ethylguaiacol were evaluated using a HSPME (headspace-solid-phase microextraction) technique and analyzed by GC. The fiber used was: divinyl-benzene/carboxen/polydimethylsiloxane (DVB/CAR/PDMS), 50 to 30 μm, Stable Flex/SS, 1 cm (Supelco, Bellefonte, PA, USA). The sample was prepared as follows: 5 mL of wine was placed into a 10-mL vial with 2.5 g of NaCl and a magnetic stirrer. The samples, after equilibration for 10 min at 25 °C, the DVB/CAR/PDMS fiber was inserted through the vial septum and exposed for 40 min at 55 °C. The 3-octanol was used as an internal standard (1.6 mg/L). A GC-2014 gas chromatograph (Shimadzu, Kyoto, Japan) equipped with a flame ionization detector and a glass Supelcowax-10 column (60 m × 0.32 mm × 0.25 mm) from Supelco was used. GC conditions: carrier gas (flow rate of 3.74 mL/min); split/splitless modality: 60 s splitless; injection and detector temperature, 230 °C. The column program was: 50 °C for 1 min, and increased 2 °C/min to 200 °C and maintained at 200 °C for 20 min. The compounds were identified and quantified by comparison with internal calibration curves of known compounds. After 60 days of treatment of the wine with the D15 formulation, the viable plate counts were carried out on the Yeast Mold Agar. 

### 4.8. Statistical Analyses

Experimental data were reported as the mean values ± standard deviations or error bars. Regarding viable cell counts, data were subjected to analysis of variance (ANOVA). The significant differences were determined using Duncan tests with associated *p*-values < 0.05. 

## Figures and Tables

**Table 1:** Determination of glucose, total protein content and diameter of inhibition halo of free-cell supernatant and broth 10 × partially purified, as middle downstream process, and D2, D15 and D18 lyophilized melted in water. Data regarding glucose and total protein contents were reported as mean values ± standard deviations. The killer toxin activity was evaluated against the *Brettanomyces bruxellensis* sensitive strain.

	Samples								
	Free-Cell Supernatants			Broth 10 × Partially Purified			Lyophilized (Stock 100 g/L)		
Determinations	Pikt	Kwkt	WA18	Pikt	Kwkt	WA18	Pikt	Kwkt	WA18
Glucose content (g/L)	2.10 ± 0.06	<0.20 ± 0.00	2.50 ± 0.20	1.00 ± 0.00	<0.20 ± 0.00	0.74 ± 0.20	0.03 ± 0.00	0.50 ± 0.02	0.03 ± 0.00
Total protein content (g/L)	8.14 ± 0.49	11.40 ± 0.04	7.98 ± 0.22	18.10 ± 0.22	8.90 ± 0.06	16.40 ± 0.14	47.60 ± 0.15	43.0 ± 0.82	47.10 ± 0.74
Halo diameter (mm)	15	23	14	18	44	17	20	44	20

**Table 2:** Growth inhibition halo of sensitive *Brettanomyces bruxellensis*. Data represent the growth inhibition halo of the sensitive yeast strain subjected to different concentrations of each killer toxin during MIC trial.

Killer Toxins	Halo Diameter (mm)				
	1 mg/mL	0.5 mg/mL	0.1 mg/mL	0.05 mg/mL	0.01 mg/mL
D2	13	10	9	0	0
D15	22	18	12	9	0
D18	11	9	0	0	0

**Table 3:** *B. bruxellensis* viability, detected with imaging cytofluorimetric assay and by viable cell counts, after 96 h of incubation with each killer toxin (1 mg/mL). The population of the sensitive yeast strain was represented as live, dead and partially PI positive cells for imaging cytofluorimetric assay, while the same populations were represented as relative percentage of live and dead cells by viable cell counts. C- represents the negative control where any toxins was added; C+ represents the positive control where the cells were killed with a treatment with 80% ethanol for 2 h.

	**Imaging Cytofluorimetric Assay**			**Viable Cell Counts**	
**Samples**	**Live Cells (%-PI)**	**Partially PI Positive Cells (%pPI)**	**Dead Cells (%+PI)**	**Live Cells (%)**	**Dead Cells (%)**
C-	70.90	0.98	28.00	100.00	0.00
D2	23.70	2.70	73.30	0.72	99.28
D15	2.06	30.10	64.10	0.02	99.98
D18	54.80	0.78	40.70	1.21	98.79
C+	0.76	0.21	98.70	0.00	100.00

**Table 4:** Origin and biochemical properties of killer toxins.

Yeast Species	Yeast Code	Killer Toxins Produced	Molecular Weight	Biochemical Properties	References
*Kluyveromyces wickerhamii*	DiSVA15	Kwkt	72 KDa	β-1,6-glucosidase activity	[14,15]
*Wickerhamomyces anomalus*	DiSVA2	Pikt	8 KDa	ubiquitin-like protein	[15,31]
*Wickerhamomyces anomalus*	DiSVA671	WA18	31 KDa	UDP-glycosyltransferase protein	[13]

## Data Availability

The data presented in this study are available in article.
